# A microfluidic model of human brain (μHuB) for assessment of blood brain barrier

**DOI:** 10.1002/btm2.10126

**Published:** 2019-01-13

**Authors:** Tyler D. Brown, Maksymilian Nowak, Alexandra V. Bayles, Balabhaskar Prabhakarpandian, Pankaj Karande, Joerg Lahann, Matthew E. Helgeson, Samir Mitragotri

**Affiliations:** ^1^ John A. Paulson School of Engineering and Applied Sciences Harvard University, 29 Oxford St. Cambridge MA 02138; ^2^ Wyss Institute of Biologically Inspired Engineering, Harvard University 3 Blackfan Circle, Boston MA 02115; ^3^ Dept. of Chemical Engineering University of California Santa Barbara CA 93106; ^4^ Biomedical Technology CFD Research Corporation Huntsville AL 35805; ^5^ Dept. of Chemical and Biological Engineering Rensselaer Polytechnic Institute 110 8th Street, Troy NY 12180; ^6^ Dept. of Chemical Engineering University of Michigan Ann Arbor MI 48109; ^7^ Dept. of Material Science & Engineering University of Michigan Ann Arbor MI 48109; ^8^ Dept. of Macromolecular Science & Engineering University of Michigan Ann Arbor MI 48109; ^9^ Dept. of Biomedical Engineering, and Biointerfaces Institute University of Michigan Ann Arbor MI 48109; ^10^ Biointerfaces Institute University of Michigan Ann Arbor MI 48109

**Keywords:** BBB, brain on a chip, microfluidic, organ on chips

## Abstract

Microfluidic cellular models, commonly referred to as “organs‐on‐chips,” continue to advance the field of bioengineering via the development of accurate and higher throughput models, captivating the essence of living human organs. This class of models can mimic key in vivo features, including shear stresses and cellular architectures, in ways that cannot be realized by traditional two‐dimensional in vitro models. Despite such progress, current organ‐on‐a‐chip models are often overly complex, require highly specialized setups and equipment, and lack the ability to easily ascertain temporal and spatial differences in the transport kinetics of compounds translocating across cellular barriers. To address this challenge, we report the development of a three‐dimensional human blood brain barrier (BBB) microfluidic model (μHuB) using human cerebral microvascular endothelial cells (hCMEC/D3) and primary human astrocytes within a commercially available microfluidic platform. Within μHuB, hCMEC/D3 monolayers withstood physiologically relevant shear stresses (2.73 dyn/cm^2^) over a period of 24 hr and formed a complete inner lumen, resembling in vivo blood capillaries. Monolayers within μHuB expressed phenotypical tight junction markers (Claudin‐5 and ZO‐1), which increased expression after the presence of hemodynamic‐like shear stress. Negligible cell injury was observed when the monolayers were cultured statically, conditioned to shear stress, and subjected to nonfluorescent dextran (70 kDa) transport studies. μHuB experienced size‐selective permeability of 10 and 70 kDa dextrans similar to other BBB models. However, with the ability to probe temporal and spatial evolution of solute distribution, μHuBs possess the ability to capture the true variability in permeability across a cellular monolayer over time and allow for evaluation of the full breadth of permeabilities that would otherwise be lost using traditional end‐point sampling techniques. Overall, the μHuB platform provides a simplified, easy‐to‐use model to further investigate the complexities of the human BBB in real‐time and can be readily adapted to incorporate additional cell types of the neurovascular unit and beyond.

## 1 INTRODUCTION

The blood–brain barrier (BBB) is the prominent barrier at the interface of the blood stream and the central nervous system (CNS) and is primarily responsible for maintaining brain homeostasis and protecting the CNS from harmful foreign entities.^1^ As the brain's first line of defense against solutes and particulates in the blood, the brain microvascular endothelial cells form a tight barrier that limits the transport of nutrients and other molecules into and out of the CNS space. Combined with pericytes and astrocytes, these cells collectively form a neurovascular unit, contributing to the overall BBB phenotype. The brain endothelium is characterized by expression of tight junction complexes lack of fenestrations, and low pinocytic activity.^2^, ^3^ Although these characteristics are imperative for normal brain function, the BBB limits the penetration of therapeutics into the brain.^4^ As a result, there is a clear need for the development of adequate models to further investigate the mechanisms of transport across the BBB in order to design better brain delivery strategies.

Assessing transport of nanoparticles, proteins, and other therapeutics across the BBB can be challenging; nonetheless, researchers have designed various in vivo models to investigate this transport in both heathy and diseased BBB.^5^, ^6^, ^7^, ^8^ Animal models inherently include all contributing factors that dictate the transport across the BBB. However, translating findings from rodent models to humans remains a challenge.^9^, ^10^ Further, the complexity of the in vivo environment also poses a challenge for interpreting the results. For example, transport of nanoparticles into the brain in vivo is a combined outcome of immune clearance and permeation across the BBB, thus making it difficult to deconvolute the contributions of each factor from the measured experimental outcome. Common techniques to investigate BBB transport of therapeutics in vivo include single carotid injections, internal carotid artery perfusion, and intravenous injections.^11^, ^12^ Using intravenous injections can be disadvantageous for investigating BBB transport due to the potential rapid metabolism of the therapeutic, resulting in metabolism‐induced artifacts and greater likelihood of clearance before reaching the brain microcirculation. Alternatively, single carotid injections and internal carotid artery perfusions can reduce the likelihood of clearance while also limiting metabolic events within the brain microcirculation. Unfortunately, these techniques are labor‐intensive, requiring significant training and expertise to properly implement.^13^ As a result, there continue to be strong interests in developing simple yet physiologically relevant in vitro models of the human BBB that are also highly tunable and customizable to be used as tools to further investigate brain‐related phenomena.

To date, the primary in vitro tool of choice for researchers studying human BBB permeability is the static transwell migration assay, also referred to as the Boyden chamber assay. These assays offer the flexibility to conduct both monolayer^14^ and coculture experiments,^15^, ^16^, ^17^ can noninvasively estimate barrier permeability using transendothelial electrical resistance (TEER) measurements, and are convenient for acquiring permeability information across a monolayer, including disease models.^18^, ^19^, ^20^ However, transwell inserts can be subject to increased, artificial paracellular diffusion at the monolayer perimeter by a phenomenon known as “edge effects,” especially for highly hydrophilic compounds.^21^ This erroneous effect results from incomplete coverage of the porous inserts at the monolayer perimeter due to the inability of the endothelial cells to form tight junctions along the inner wall of the apical chamber.^22^ Typically, analyte concentrations are sampled from the apical or basolateral chamber over time without the ability to actively monitor transport. Additionally, depending on the cell culturing conditions and experimental setup, TEER values can vary significantly.^23^ To confound these reported values further, reports often misrepresent the TEER value by describing it in terms of total resistance or area‐dependent resistance.

Hemodynamic shear stress experienced by endothelial cells is an important mechanotransduction regulator not present in static transwell migration assays. Depending on the blood vessel geometry and condition, endothelial cells can experience a range of shear stresses. In vitro studies report shear stresses between <1 and 85 dyn/cm^2^ induce a variety of biological responses.^24^ For instance, shear stress acts as a pleiotropic modulator of the endothelial cell physiology, regulating genes involved in cell division, differentiation, migration, extracellular matrix protein secretion, cell–cell adhesion, and apoptosis.^25^ As a result, shear stress contributes to an overall polarized brain endothelium, influencing such properties as asymmetric expression of localized enzymes and carrier‐mediated transport systems, production of vasoactive substances and cell adhesion molecules, cell survival, and energy metabolism.^26^, ^27^, ^28^ The maintenance of brain microvascular endothelial cells is directly impacted by this hemodynamic shear stress, influencing tight junction formation and multidrug resistance transporter expression.^29^ Unlike endothelium in other organs of the body, brain microvascular endothelial cells resist elongation in response to both curvature and shear stress.^30^, ^31^, ^32^ Interestingly, a report by Garcia‐Polite et al. demonstrates cerebrovascular function (i.e., expression of tight junction proteins ZO‐1, Claudin‐5, and efflux pump P‐gp) can be directly correlated to the magnitude and nature of shear stress. Higher than physiologically relevant (40 dyn/cm^2^) and pulsatile shear stresses resulted in downregulation of ZO‐1, Claudin‐5, and P‐gp; however, tight junction marker expression recovered when physiological shear was reestablished,^33^ further suggesting the importance of maintaining hemodynamic shear stress among in vitro systems to more accurately represent the BBB microenvironment.

Recent developments in this field have resulted in a diversity of three‐dimensional cell culture models and several dynamic systems with the ability to incorporate hemodynamic shear.^34^, ^35^ Still, simultaneous visualization of the BBB and the associated transport through the barrier in real‐time remains a challenge.^36^, ^37^, ^38^, ^39^ Direct visualization at a cellular level provides real‐time monitoring of the cellular morphology and can be used as a proxy for cell behavior. This allows for measurement of protein localization information in addition to expression levels. With the ability to directly capture transport, one can collect more complex information, such as the precise interactions of a particulate of interest (e.g., monocyte, virus, nanoparticle) before, during, and after interacting with the BBB, which otherwise would be impossible. This capability also simplifies the measurement of transport kinetics while simultaneously offering higher temporal resolution than would be possible using a traditional sampling‐type approach.

Some models have attempted to visualize transport across the BBB in real time;^40^, ^41^ however, the shear stresses applied in these experiments (3.8 × 10^−3^ to 0.15 dyn/cm^2^) are often orders of magnitude lower than what are considered physiologically relevant within the brain microvasculature (1–30 dyn/cm^2^).^24^, ^42^, ^43^, ^44^ Maintaining the culture under higher shear stress for prolonged periods of time in a microfluidic environment poses a significant challenge.^45^ This limitation is especially significant given that previous reports indicate low shear stresses may be insufficient to induce the proper morphological and biochemical changes. For example, studies performed using a bovine aortic endothelial cell model have shown that expression of p53, a tumor suppressing protein, was upregulated when the cells were subjected to 3 dyn/cm^2^ but not 1.5 dyn/cm^2^. The mechanotransduction effects of shear stress are believed to mediate several cellular functions, including the inhibition of cellular proliferation by the activation of p53 expression with the potential of arresting endothelial cell apoptosis.^46^ Furthermore, in the absence of laminar flow, static monolayers can be subject to uncontrolled growth, resulting in formation of multiple layers, if allowed to proliferate.^47^ Therefore, a model with the ability to incorporate physiologically relevant shear stresses is essential to effectively capture biologically relevant transport across any barrier in direct contact with the bloodstream. Additional limitations of existing models to probe human brain permeability include the use of rodent brain endothelial cells,^36^, ^40^ which do not exhibit the same anatomical and molecular complexities as their human counterparts.^48^, ^49^ Alternatively, while the use of primary human brain endothelial cells may have significant advantages,^37^, ^50^ these cells can be difficult to acquire, variable in nature, and challenging to culture and maintain, especially in a microfluidic environment.^51^

Herein, we report the development of a microfluidic human BBB model (μHuB) with the ability to directly monitor both the barrier and associated transport in the presence of physiologically relevant shear conditions. This model leverages a commercially available chip with low required volumes and a well‐characterized, immortalized cell line to provide a convenient and effective research tool for investigating the human BBB and its permeability. Because of the transparent nature of the glass and polydimethylsiloxane (PDMS) μHuB structure, temporal and spatial permeability data across the BBB can be easily acquired using a conventional or confocal fluorescent microscope. We further demonstrate that μHuB is modular and can be readily adapted for more complex, coculture experiments to further bridge the gap between existing tools for investigating the human BBB and underlying biology.

## 2 RESULTS

### 2.1 Culture of hCMEC/D3 cells in μHuB device

**Figure 1:**
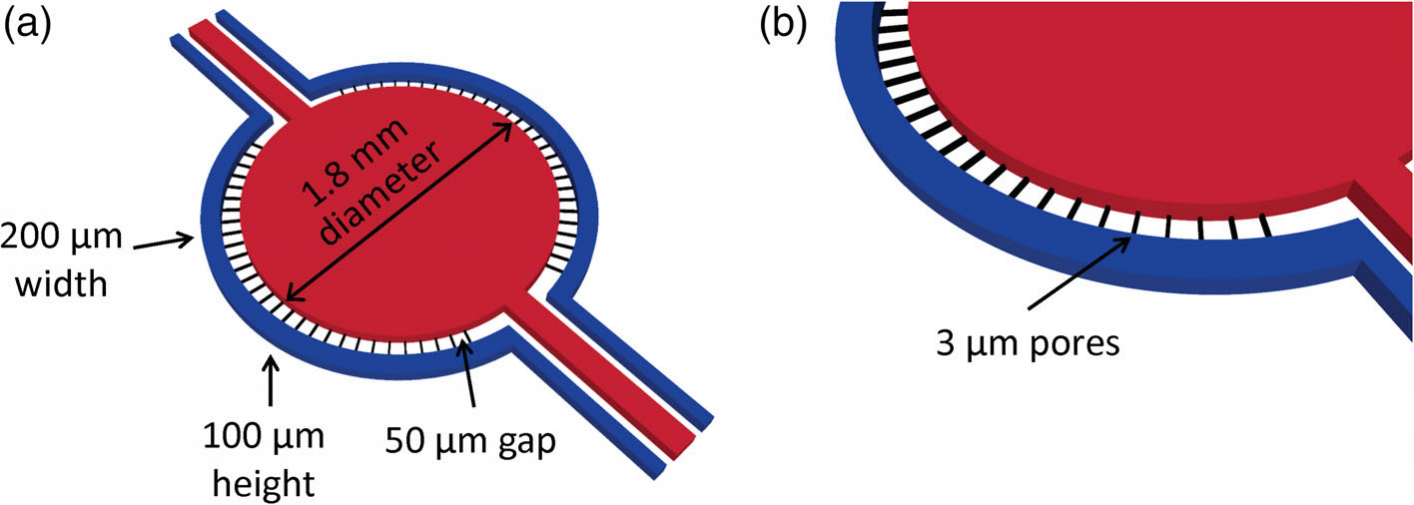
Schematic of μHuB device. μHuB consists of 2 outer, apical compartments (blue) and 1 central, basolateral compartment (red). (a) An overview of the entire μHuB layout with appropriate dimensions. Apical compartments are 200 μm (width) by 100 μm (height). Basolateral compartment is 1.8 mm (diameter) by 100 μm (height). Interconnecting channels connecting the basolateral to the apical compartments are spaced by 50 μm (width). (b) Zoomed‐in region of the apical and basolateral compartments connected by 3 μm (width) by 3 μm (height) by 50 μm (depth) pores (black)

The scaffold for μHuB is a commercial microfluidic device (SynVivo Inc.) possessing a central disk‐shaped chamber surrounded by vascular channels. The interface between the central channel and the vascular channel possesses 3 μm slits. The vascular channel has 50 μm travel distance (Figure 1). This design was chosen to facilitate comparisons with other transwell models with a pore size of 3 μm, which are commonly used to study transport across static in vitro models.^52^ Initially, devices were coated with a variety of basement membranes, including rat tail collagen Type 1, human fibronectin, and laminin, which have been used to promote cell adhesion in the literature.^40^, ^53^, ^54^ Optimal cell adhesion was observed with a thin coating of human fibronectin and was used for all studies reported. Consistent cell attachment to the upper portion of the PDMS channel proved challenging using standard injection techniques. Therefore, we adopted a two‐step seeding protocol as described by Herland and coworkers^50^ wherein the device is inverted after initial seeding and reseeded in the upright position. This resulted in confluent monolayers being reproducibly present on every surface of the channel. Confluent monolayers were formed over 24 hr, creating a well‐defined lumen, and were maintained under static conditions for a period of 3 days before being subjected to shear stress.

hCMEC/D3 cells are a commercially available, immortalized cell line that has phenotypic characteristics of human brain endothelial cells. Studies have demonstrated that hCMEC/D3 is a promising cell line for in vitro BBB experiments, often used to elucidate the functional roles of the neurovascular unit. This cell line has shown to restrict permeability to paracellular tracers, express functional P‐Glycoprotein (P‐gp) and other efflux transporters (e.g., ATP‐binding cassette transporters), undergo receptor‐mediated transport, respond to inflammatory cytokines and flow‐based shear stresses, form vasculature with an inner lumen, and express tight junction proteins (e.g., JAM‐A, Claudin‐5, ZO‐1), similar to in vivo human BBB.^55^, ^56^, ^57^, ^58^, ^59^ Thus, hCMEC/D3 exhibits the desired BBB characteristics to be used for a model of the BBB.

**Figure 2:**
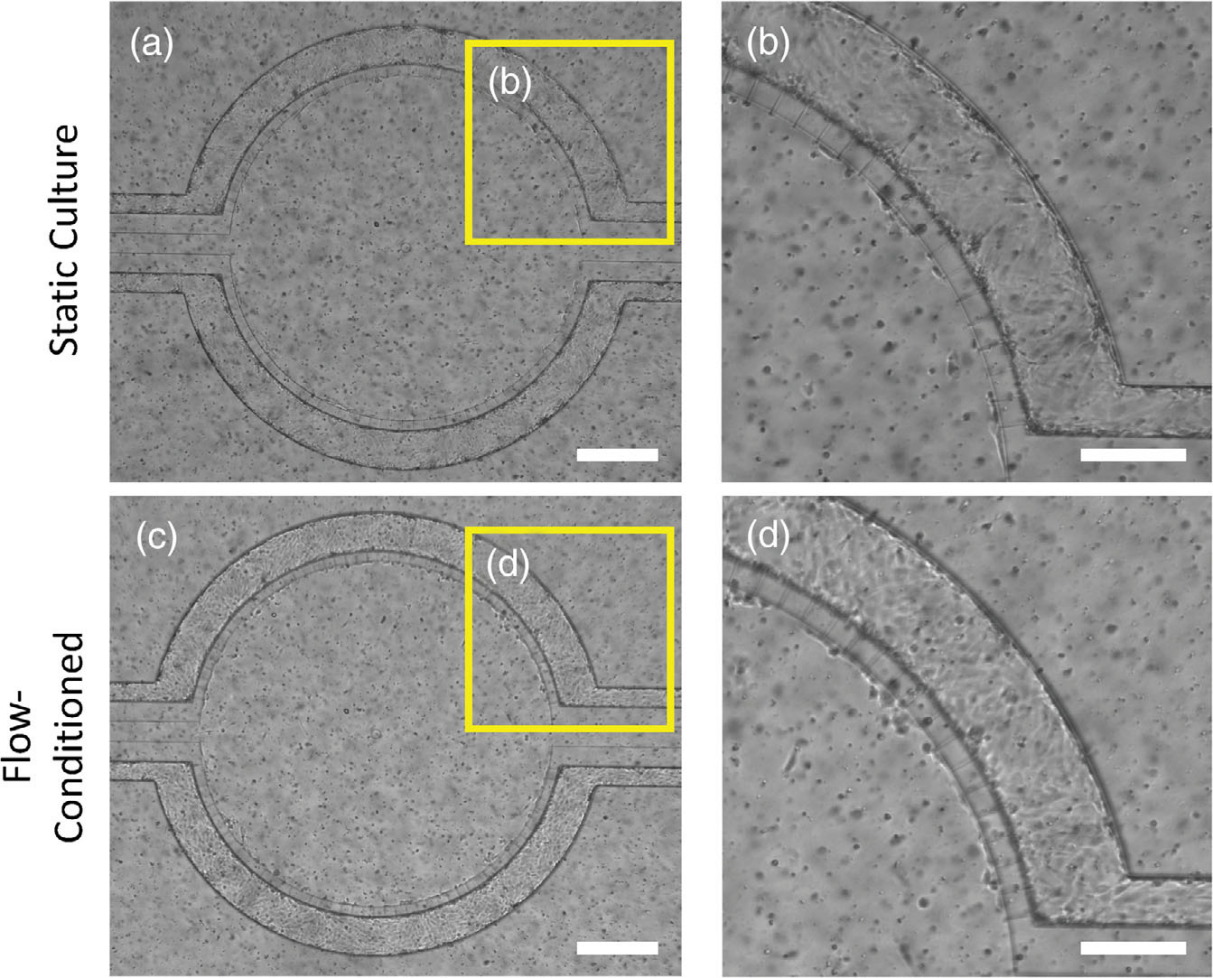
hCMEC/D3 monolayer can withstand physiological shear stresses in μHuB. Brightfield micrographs of hCMEC/D3 cells grown under static conditions for 3 days (a and b) and after conditioning to physiologically relevant shear stress (2.73 dyn/cm^2^) using a linear ramp conditioning protocol overnight (c and d). All images depict the same μHuB device at different points in time. b and d represent zoomed‐in regions of a and c, respectively, demonstrating hCMEC/D3 resistance to elongation under flow conditions and its ability to withstand these flow conditions. (scale bar for *a* and *c* = 400 μm; for *b* and *d* = 200 μm)

Sudden exposure of hCMEC/D3 cells to physiologically relevant shear stresses after monolayer formation under static conditions caused severe morphological changes, including cell shrinkage and detachment from substrate, indicating cell stress, and ultimate death. Previous reports have suggested shear stress inhibits cell proliferation and at high levels leads to death of mammalian cells.^60^, ^61^, ^62^ Therefore, we chose to initially allow the monolayers to grow statically before gradually and linearly increasing the shear stress applied to the monolayers via fluid flow over an extended period of time to condition the hCMEC/D3 cells to shear stress. To our knowledge, such an approach has not been reported as a method for ensuring brain endothelial cells can be cultured under physiologically relevant flow. The cells were first grown statically in the μHuB for a period of 3 days (Figure 2a,b). Cells were then exposed to a low shear stress (0.05 dyn/cm^2^) which was increased linearly over 12 hr to a physiologically relevant shear stress of 2.73 dyn/cm^2^ for 6 hr (Figure 2c,d). hCMEC/D3 cell morphology does not change significantly after 18 hr of being cultured in this manner (12 hr of ramping and an additional 6 hr of flow at 2.73 dyn/cm^2^). Cells retain this morphology for over 24 total hours under flow, demonstrating the effectiveness of this ramping protocol in conditioning the monolayers to survive realistic flow conditions, thereby recapitulating an essential aspect of the BBB in vitro.

### 2.2 Characterization of μHuB structure

**Figure 3:**
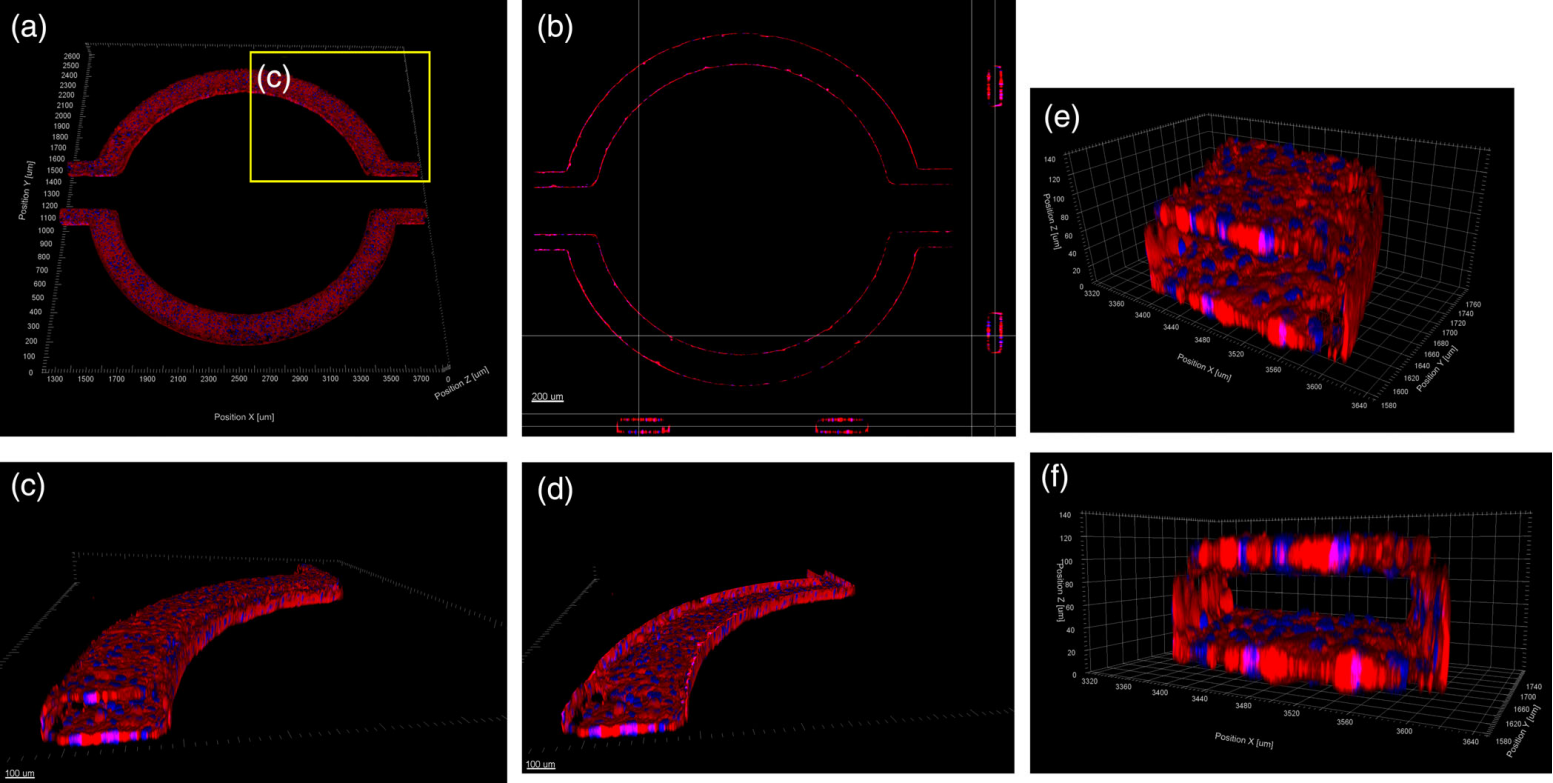
hCMEC/D3 forms a complete inner lumen in μHuB. (a–f) Confocal images of hCMEC/D3 monolayers in the μHuB after conditioning to flow stained with ActinRed™ 555 ReadyProbes™ (actin, red) and Hoechst 33342 (nucleus, blue). (a) Onward‐looking view of μHuB device consisting of two vascular (apical) compartments lined with hCMEC/D3 monolayers. (b) Cross‐sectional view of hCMEC/D3 monolayers in μHuB forming a complete inner lumen approximately 200 μm (width) by 100 μm (height). (c) Onward‐looking view of one quadrant of the μHuB model as outlined in yellow in (a). (d) Lower half of (c), lined with a complete hCMEC/D3 monolayer. (e) Cross‐sectional view of inner lumen. (f) Same cross‐section as (e) at 90° viewing angle

**Figure 4:**
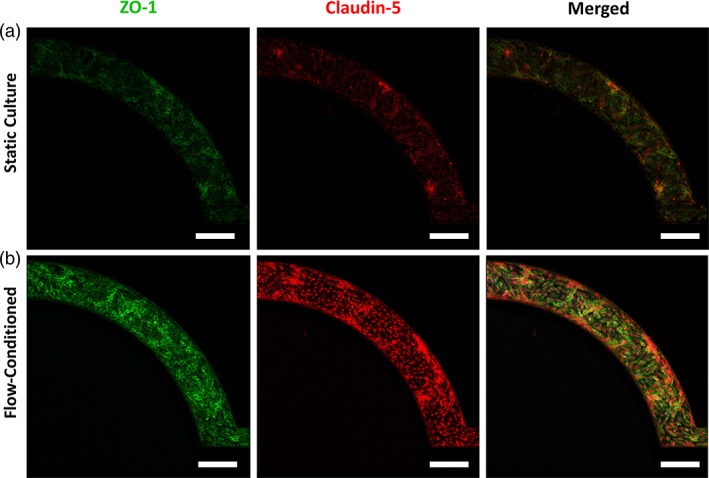
Phenotypic expression of tight junction proteins in μHuB. The hCMEC/D3 monolayers in μHuB express tight junction proteins (ZO‐1, green & Claudin‐5, red) under (a) static culture for 3 days and (b) when conditioned to physiologically relevant flow (2.73 dyn/cm^2^) using a linear ramp conditioning overnight protocol. Expression of both ZO‐1 and Claudin‐5 increased in response to the fluid flow. (scale bars = 200 μm)

hCMEC/D3 cells form both a confluent monolayer and complete lumen in the μHuB, as would be expected in vivo. Monolayers were fixed after ramping and stained with an actin stain and nuclear dye prior to being imaged with a confocal microscope (Figure 3). Cells formed a complete lumen lined by a confluent monolayer on the bottom, sides, and top of the microfluidic channels, resembling an in vivo BBB (Figure 3b). This is exemplified by the three‐dimensional reconstructions of the μHuB, where one section of the complete vascular compartment (Figure 3c) is sectioned in half (Figure 3d). Monolayers line the complete inner channels of this microfluidic device, forming an inner lumen which allows for media and other components to flow through (Figure 3e,f). Expression of tight junction proteins is critical for a realistic model of the BBB. Previous work has shown that the hCMEC/D3 cell line expresses two of the most relevant tight junction proteins, Claudin‐5 and ZO‐1 in traditional cell culture conditions.^55^, ^57^, ^63^ Therefore, antibody staining for Claudin‐5 and ZO‐1 was performed after culturing 3 days statically (Figure 4a) and after conditioning to physiologically relevant fluid flow (2.73 dyn/cm^2^) (Figure 4b). The diffuse expression profiles of these tight junction markers were characteristic of other traditional static reports.^64^ Within the μHuB, the magnitude of the expression of these proteins, however, increased dramatically in response to fluid flow as compared to its static counterpart.

**Figure 5:**
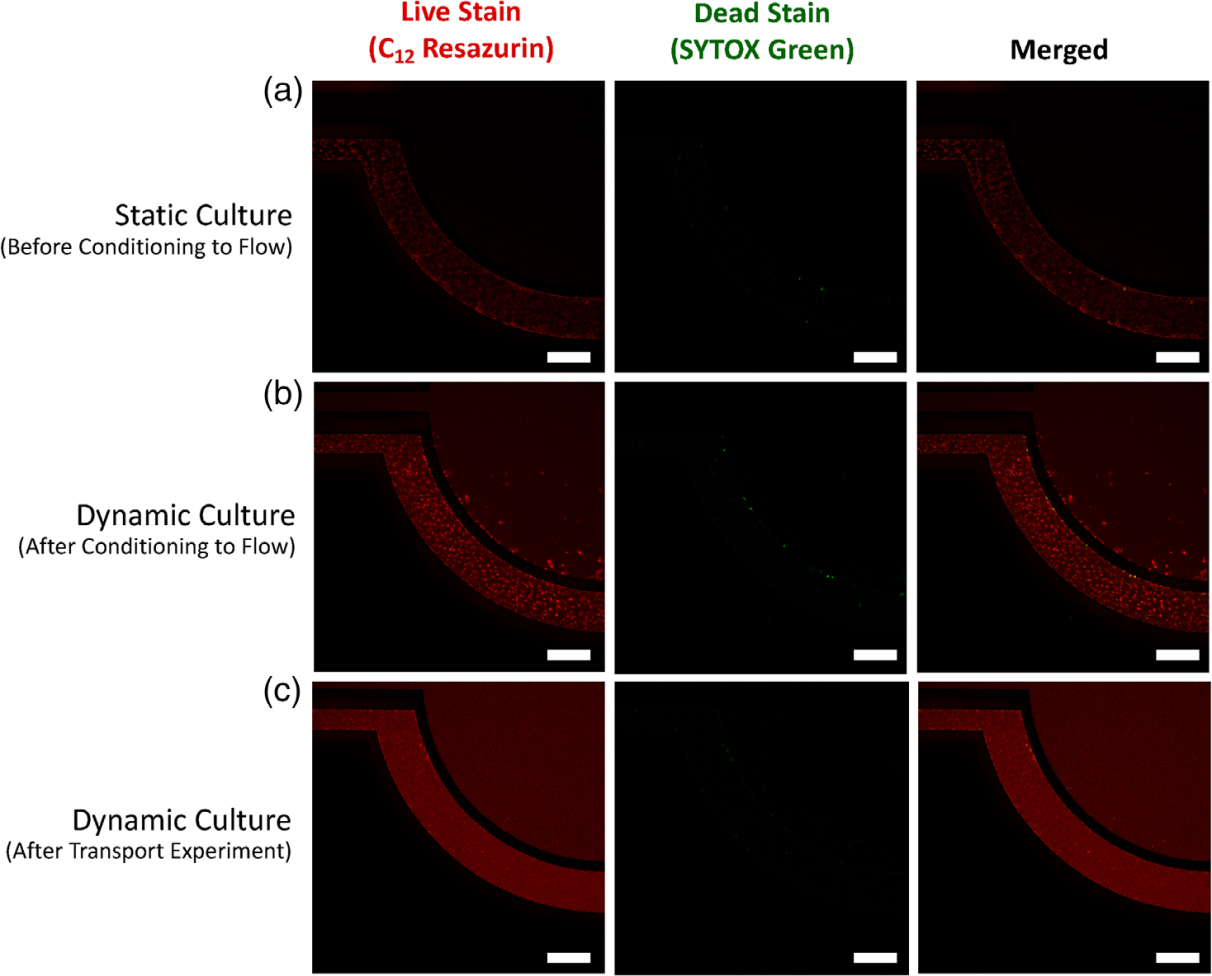
hCMEC/D3 monolayers remain viable during dynamic culture before and after analyte transport. hCMEC/D3 monolayers in μHuB remain metabolically active as demonstrated by high levels of red, C_12_‐resazurin (alive) fluorescence with negligible expression of green, SYTOX fluorescence (injured) after (a) static culture for 3 days (b) after conditioning monolayers overnight using the linear ramping protocol to 2.73 dyn/cm^2^ and (c) after conducting a transport experiment using nonfluorescent dextran 70 kDa. (scale bars = 200 μm)

The impact of shear stress on cell viability was investigated with a live/dead assay. Cell viability was measured by the reduction of C_12_‐resazurin to red‐fluorescent C_12_‐resorufin. SYTOX Green was used as a counterstain to identify cells with compromised cell membranes. This green‐fluorescent nucleic acid stain cannot penetrate intact cell membranes and remains non‐fluorescent until bound to the nucleus. Relative intensities of red and green fluorescence can be used to identify live cells from injured or dead cells, respectively. hCMEC/D3 cells grown in the microfluidic device exhibited high cell viability and negligible cell death or injury both before (Figure 5a) and after (Figure 5b) conditioning to shear stress, indicating the monolayers are viable for extended periods of time under shear stress using the described ramping protocol.

### 2.3 Permeability of FITC‐Dextrans across μHuB

To quantitatively assess the barrier permeability from the apical to basolateral side of the μHuB model, fluorescently labeled dextrans of different molecular weights (10 and 70 kDa) were used as probes having approximate Stokes' radii of 23 and 60 Å, respectively, as provided by the manufacturer. Constant molarity (312.5 nM) of the tracers within the apical chamber was maintained for all experiments to investigate how size directly impacted the permeability of the μHuB model. For acellular scaffolds devoid of a cellular barrier, 10 and 70 kDa FITC‐dextrans experienced high permeabilities (*P*_scaffold_) of 5.0 × 10^−5^ and 3.1 × 10^−5^ cm/s, respectively. By subtracting the acellular scaffold permeability (*P*_scaffold_) from the total permeability observed when the cellular barrier was present (*P*_total_) as described by Equation 2, the permeability of the endothelial barrier (*P*_e_) was determined for 10 kDa FITC‐dextran (15 × 10^−6^ cm/s) and 70 kDa FITC‐dextran (3.7 × 10^−6^ cm/s). Overall, the relatively high *P*_scaffold_ values combined with the low *P*_total_ values indicate that transport of the FITC‐dextrans in the μHub was limited by diffusion through the cellular barrier rather than the scaffold architecture. The reported *P*_e_ values correlate well in both trend and magnitude with in vivo transport modeling of macromolecules across the BBB.^65^

**Figure 6:**
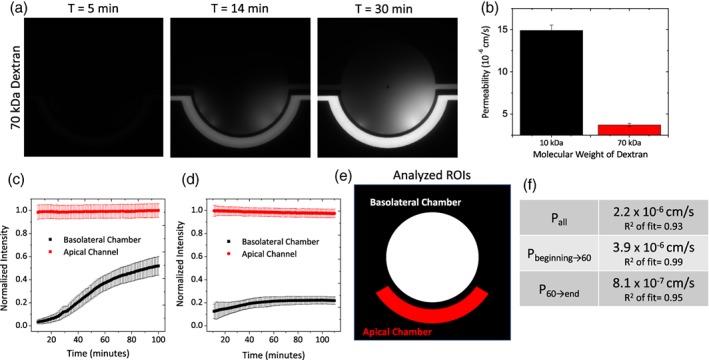
Real‐time permeability assessments of FITC‐dextran with μHuB. (a) Representative images of 70 kDa dextran penetration through the microfluidic BBB. (b) Calculated cellular permeability (*P*_e_) of various molecular weight dextrans through the microfluidic BBB model. Permeability of the acellular scaffold (*P*_scaffold_) was subtracted from the overall permeability observed (*P*_total_) to determine the permeability of the cellular barrier (*P*_e_). Error bars represent 95% confidence interval. (c) Example normalized intensity profiles of transport for a single device with 10 kDa dextran tracer. Error bars represent *SD*. (d) Example normalized intensity profiles of transport for a single device with 70 kDa dextran tracer. Error bars represent *SD*. (e) Analyzed regions of interest for (c and d). (f) Permeabilities calculated from (d) based on the inclusion of different temporal regions of the intensity profile as well as the *R* ^2^ value of the fit

With the added ability to observe transport in real time within the μHuB, localized permeabilities can be calculated for precise temporal regions of interest. Using the representative data in Figure 6d, the intensity profile could exhibit three regions with different slopes: a relatively rapid increase at early time points, a more gradual increase at later time points, and a final plateau region. By calculating the inflection points with higher order fits, the described transitions between these regions occur at approximately *t* = 60 min and *t* = 90 min for the inflection and plateau, respectively. Fitting different slopes on either side of the inflection point results in a more precise fit of the data compared to using a single slope for the profile (Figure 6f).

To assess the vitality of the monolayers in the μHuB after a transport experiment, cell viability of the monolayers in the μHuB was investigated on shear stress‐conditioned cells after 6 hr of constant flow with cell culture medium and after an additional 3 hr of constant flow with a nonfluorescent 70 kDa dextran solution (312.5 nM) to ensure no fluorescence interference with the viability assay resulted. As demonstrated in Figure 5c, monolayers continued to exhibit high cell viability and low cell toxicity, indicating the largest molecular weight dextran solution had no significant impact on the viability of the μHuB model over the observed time periods and at this concentration.

### 2.4 Expansion of the μHuB model with astrocytes

**Figure 7:**
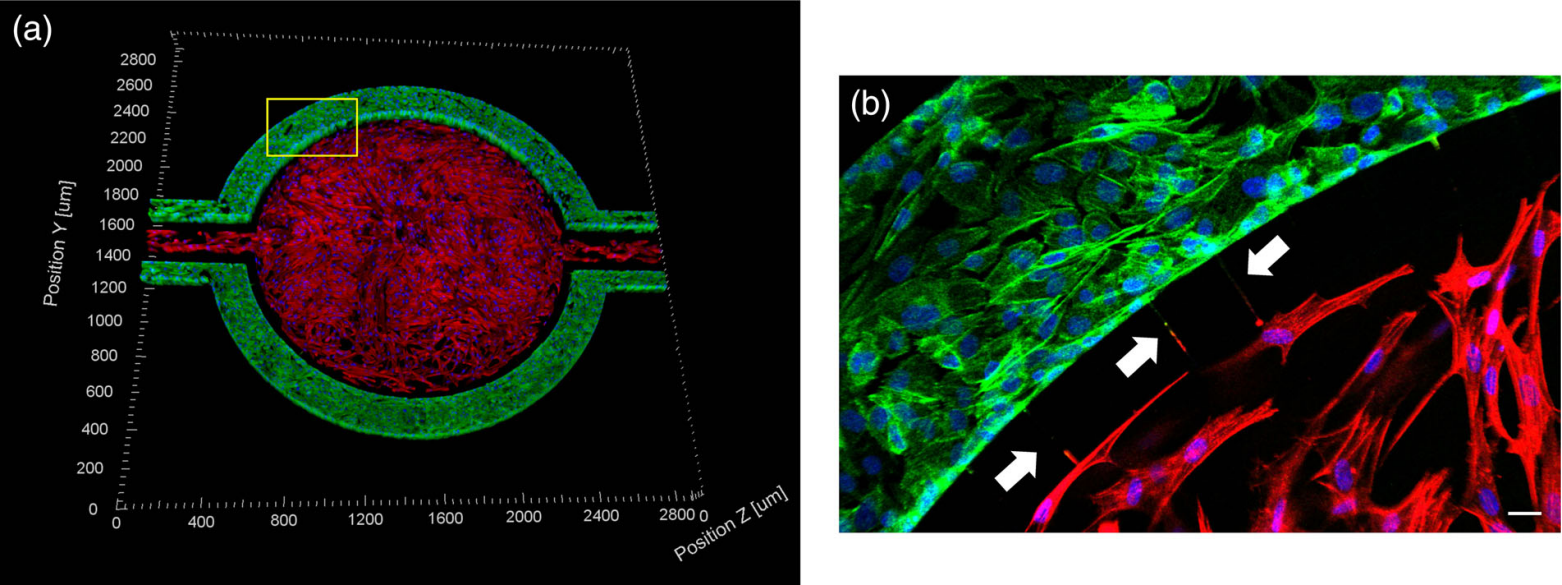
Coculture of hCMEC/D3 and primary human astrocytes in μHuB. hCMEC/D3 monolayers (green) were cultured in the vascular (apical) compartments with primary human astrocytes (red) in the tissue (basolateral) compartment (nuclei, blue). (a) Onward‐looking view of complete, three‐dimensional reconstruction of the coculture μHuB. (b) Zoomed‐in yellow region of (a) with arrows pointing to regions where astrocyte end‐feet are protruding to hCMEC/D3 monolayer. (scale bar for *b* = 20 μm)

As the neurovascular unit comprising the BBB contains additional cell types beyond the brain endothelium, μHuB can be expanded by coculturing additional cells in the central compartment to further investigate how transport and other cellular functions are affected in the presence and/or absence of specific cell types. As proof of concept, primary human astrocytes were seeded into the central compartment, lined with a thin coating of Matrigel to facilitate the cellular attachment. This coculture device (Figure 7) was maintained over the same time period with the brain endothelial cells and cultured as described for the simpler, endothelial cell only μHuB model. Several connecting channels between the apical and basolateral compartments of the device appear to have astrocyte end‐feet protruding through the basolateral compartment and interacting with the hCMEC/D3 monolayer, regions highlighted by white arrows (Figure 7b).

### 2.5 Discussion

We have presented the design and characterization of a realistic yet simple in vitro model of the human BBB: μHuB. Importantly, the μHuB recapitulates several of the most critical aspects of the in vivo BBB, specifically the incorporation of appropriate brain endothelial cells^59^ into a vessel‐like architecture that exposes the cells to shear.^66^ Moreover, by combining a commercially available, immortalized cell line with a straightforward, commercially available microfluidic chip, we have developed a highly accessible model that can be readily adopted and utilized as an experimental tool and analysis method for dynamically visualizing particulates of interest in future studies.

An essential, functional participant of the neurovascular unit is the basement membrane. Basement membrane in the brain is primarily composed of laminins and collagen IV.^67^ We found, however, that hCMEC/D3 cellular morphology and adherence to the internal glass and PDMS surfaces were optimal when coated with human fibronectin. This may be partially attributed to the structural support provided by the chip itself, as collagen IV has been implicated to have a primarily structural, scaffold‐like function.^68^ Future studies should investigate how different basement membranes and combinations thereof contribute to overall barrier integrity and function.

Immortalized cells are imperfect mimics of their primary cell precursors. Prior work,^40^, ^69^ as well as the work reported herein, demonstrate the ability of immortalized cells to withstand shear stresses for extended periods of time. We therefore hypothesize these cells have not completely lost their ability to survive under shear. Thus, by gradually increasing the shear in a linear manner, the hCMEC/D3 cells were able to survive under increased shear well over 12 hr. Cells remain adhered to the surface and retain their morphology (Figure 2). For further validation of cell survival under physiologically relevant shear, cell viability was assessed both before (Figure 5a) and after (Figure 5b) the conditioning protocol. Negligible injured signal in both cases indicated cell membranes have not been compromised while the live signal remained strongly expressed. As the dead or injured signal comes from C_12_‐resazurin reduction, which occurs in the mitochondria, this reduction directly correlates to metabolic activity and can be quantitative (i.e., a higher signal is indicative of more metabolic activity). As a result, the metabolic rates of the monolayers are not negatively impacted while under shear in such a manner to cause significant cellular toxicity.

Confirming the formation of a cellular model with a complete inner lumen is challenging using a conventional light microscope. Therefore, the flow‐conditioned model was fixed, stained, and imaged via confocal microscopy. Cells completely lined the bottom, sides, and top of the apical channel in the device without any regions devoid of cells (Figure 3). These images further indicate the effectiveness of the conditioning protocol and the structural integrity of the monolayer, clearly forming a cellular barrier between the outer and inner compartments. Complete coverage of the apical compartment surface is vital to accurately quantify the transport through an intact barrier, which can be challenging for transwell models due to “edge effects.”^70^

A prominent characteristic of the blood brain barrier is the high expression of specific tight junction markers (e.g., Claudin‐5 and ZO‐1), forcing most particulates to undergo a transcellular route of transport.^71^ hCMEC/D3 monolayers grown statically and flow‐conditioned within the device were stained for Claudin‐5 and ZO‐1. Protein expression remained intact both before and after flow‐conditioning, indicating that our flow‐conditioned model conserved tight junction expression similar to an in vivo BBB (Figure 4).

The functional properties of our model were investigated by conducting permeability experiments using dextrans of varying molecular weights. These and other tracer compounds, like Evans blue and horseradish peroxidase, are commonly used to assess the permeability of the BBB. 72, 73 The tight intercellular junctions between brain endothelial cells has been shown to exclude passive transport of molecules having Stokes' radii >10 Å. 74, 75, 76 As in vitro models do not fully recapitulate all of the necessary components for such a “tight” BBB, researchers often use dextrans with varying Stokes' radii to determine the relative “leakiness” due to passive diffusion around the endothelial cells. As expected, a size‐dependent trend was observed in the permeability (*P*_e_), where molecules with a larger Stokes' radii crossed the barrier at a reduced rate (Figure 6).

Overall, transport of the tracers reported were comparable in magnitude to those measured in prior experimental work using neonatal rat brain endothelial cells on a similar scaffold design (15 × 10^−6^ cm/s for a 10 kDa dextran reported here versus 40 × 10^−6^ cm/s for a larger 40 kDa dextran reported by Deosarkar and coworkers).^40^ Our permeability data also agree with mathematical modeling to calculate permeability values for macromolecules with similar Stokes' radii across an endothelial barrier.^65^ Yuan et al. measured permeabilities for FITC‐dextrans (10 and 70 kDa) in vivo. Both dextrans were found to exhibit low, but detectable permeabilities of 0.31 × 10^−6^ cm/s for 10 kDa and 0.15 × 10^−6^ cm/s for 70 kDa.^77^ These values are much lower than our reported findings as well as for other in vitro models. One explanation for this could be that as hCMEC/D3 cells are an immortalized cell line, tight junction expression may be reduced as compared to their primary counterparts. Researchers have developed a variety of different human brain endothelial cell lines, including BB19, hBMEC, hCMEC/D3, and TY10. Eigenmann and coworkers report dramatic differences between the tight junction protein expressions between these immortalized cell types.^63^ Theoretically, the use of primary human brain microvascular endothelial cells in the μHuB model would lead to a reduction in the permeability. Inclusion of additional cellular components (e.g., astrocytes and pericytes) may also enhance the barrier properties. Sajja and coworkers^78^ as well as Herland and coworkers^50^ have shown that the addition of these other cell types caused a reduction in the permeability values. Modeling by Li and coworkers suggests that the astrocytes contribute significantly to the diffusive barrier properties of the BBB.^65^

The permeabilities reported in our study were calculated based on our current understanding of small macromolecule translocation across the BBB, namely that the transport of dextran tracers through the BBB should remain constant with time. The transport data acquired using the μHuB can also be used to investigate potential temporal differences in permeability. As seen in Figure 6f, different temporal regions of a single experiment can have apparent permeabilities that differ over twofold but are still comparable to previously reported literature. To our knowledge, these differences are unlikely to be captured using other tools. With the dynamic visualization capability of the μHuB, heterogeneities originating from spatial biological variability can also be assessed in a single experiment by analyzing the local permeability at different azimuthal locations along the semipermeable barrier. To our knowledge, investigations into this type of variability have not been reported to date. As a result, the μHuB can be a powerful tool for developing a deeper mechanistic understanding of any type of particulate transport through the BBB both in time and space.

As the blood–brain barrier consists of various cell types in addition to brain endothelial cells, including astrocytes, pericytes, and glial cells, a coculture of primary human astrocytes and hCMEC/D3 was successfully cultured using a similar protocol for the hCMEC/D3 only models to achieve complete lining of the central compartment with primary astrocytes. Different cell types can easily be incorporated into the central compartment to further investigate the functional roles of BBB components and how specific cell‐to‐cell interactions affect transport of molecules across the brain endothelium. Additionally μHuB can be easily expanded to incorporate additional components of interest, including the use of differentiation factors (e.g., 8‐CPT‐cAMP and Ro 20–1,724),^79^ primary human brain endothelial cells instead of the immortalized line, modification of cell type ratios to represent different regions of the brain,^80^ and modulation of the applied shear stress, to create a holistic model of a healthy BBB. μHuB can also be readily modified to further investigate how transport is affected in a diseased state, such as when there is inflammation caused by a traumatic brain injury or as the result of an invasive glioblastoma.

### 2.6 Conclusions

We have reported the development of μHuB, an easy‐to‐use human microfluidic blood–brain barrier model. The ability of endothelial monolayers in the μHuB to mimic the lumen of the BBB depends critically on a newly developed protocol to condition the cells to physiologically relevant shear conditions. Using this conditioning protocol, monolayers can be maintained at physiologically relevant shear stresses to spatially and temporally resolve the transport of particulates across the BBB in real‐time. We anticipate that experiments in the μHuB can easily be expanded to quantify and mechanistically investigate transport of molecular and particulate species across various states of the BBB.

## 3 MATERIALS AND METHODS

### 3.1 μHuB device architecture

The idealized coculture microfluidic devices used in this study were obtained from SynVivo, Inc. (Huntsville, AL). The devices consisted of a central (basolateral) compartment, encompassed by an outer (apical) compartment. The central and outer compartments were separated by PDMS pillars with 3 μm slits, creating a barrier region between the outer and inner compartments (See Figure 1 for device schematic). The outer compartment was lined with brain endothelial cells and experienced perfusion similar to physiological fluid flow conditions.

### 3.2 Cell culture

The immortalized human cerebral microvascular endothelial cell line (hCMEC/D3) was obtained from Millipore Sigma and maintained with EndoGRO‐MV Complete Culture Media Kit supplemented with 1 ng/mL human animal‐free basic fibroblast growth factor (bFGF‐AF) and 1% Penicillin–Streptomycin. Cells were cultured on collagen‐coated tissue culture flasks coated with 1:20 dilution of Corning® Collagen Type I, Rat Tail, which was allowed to coat in the incubator for 1 hr prior to use. Cells were incubated at 37 °C, 95% humidity and 5% CO_2_ until confluent. Cells were used between passage 27 and 36.

For coculture experiments, primary human astrocytes (Catalog #1800) were obtained from ScienCell and maintained astrocyte medium (Catalog #1801) also obtained from ScienCell. Cells were cultured on poly‐L‐lysine coated tissue culture flasks (2 μg/cm^2^), which were allowed to coat in the incubator overnight prior to use. Cells were incubated at 37 °C, 95% humidity and 5% CO_2_ until confluent.

### 3.3 Culture of hCMEC/D3 and primary astrocytes in μHuB

To facilitate endothelial cell attachment, human fibronectin (300 μg/mL) was injected in the outer compartment and allowed to incubate for 1 hr at 37 °C and 5% CO_2_. The entire device was perfused with complete cell culture media. To devoid the device from any residual entrapped air, the device was primed using inert N_2_ gas at 6 PSI for 30 min. Devices were placed inside cell culture incubator prior to use. For coculture experiments, the device was first perfused with a thin‐coating of Matrigel (1:5) in the central compartment for 1 hr at 37 °C and 5% CO_2_ prior to coating the outer channels with human fibronectin (300 μg/mL) as described previously.

hCMEC/D3 grown to 70 to 80% confluency were trypsinized and resuspended in cell culture media with increased serum concentration (10%). Cell suspension at ~5 × 10^7^ cells/mL was injected into the outer compartment at 6 μL/mL using a Harvard Apparatus Pump 11 Pico Plus Elite and placed inside the incubator upside down to facilitate attachment to the upper PDMS regions of the channel. After sufficient cellular attachment, an identically seeded flask of hCMEC/D3 cells was trypsinized, and cells were seeded with the device in the upright position. Following cellular attachment, μHuB was perfused with complete cell culture media at 5 μL/min. Cells were fed daily by perfusion of the device with cell culture media containing 10% FBS for the first day after seeding, and 5% FBS media for each subsequent day.

For coculture seeding, after replenishing media in the outer compartments containing endothelial cells, primary human astrocytes were injected into the central compartment and allowed to attach.

To condition cells to physiological shear stresses, 10% FBS containing media was injected according to a linear ramp profile (100 μL/min–5 μL/min) over 12 hr using a Harvard Apparatus PHD ULTRA™ with a 6 × 10 MultiRack attachment for multi‐syringe perfusion. Constant 5 μL/min injection rate was maintained for at least 6 hr prior to use. Devices were inspected for any bubble formation and immediately used for further studies.

### 3.4 Visualization and inner lumen characterization of μHuB with actin stain

After flow conditioning of model, DPBS was perfused to replace the cell culture media. 4% PFA was injected into all device compartments and allowed to remain at room temperature for 15 min. The device was again perfused with DPBS to move any residual PFA. Fixed cells permeabilized using 0.2% Triton X‐100 in DPBS for 10 min. The device was again perfused with DPBS to move any residual Triton X‐100. Thermofisher ActinRed™ 555 ReadyProbes™ Reagent was used to stain for cytoskeleton, using two drops per mL of DPBS for 30 min at room temperature. The device was perfused with DPBS one final time prior to imaging.

For coculture μHuBs, the same actin staining procedure described above was used with slight modifications. ThermoFisher ActinGreen™ 488 ReadyProbes™ was used to stain hCMEC/D3 cytoskeleton in the vascular compartment and Thermofisher ActinRed™ 555 ReadyProbes™ Reagent was used to stain primary human astrocyte cytoskeleton in the tissue compartment. For each dye solution, two drops per mL of DPBS was used and allowed to remain in the respective compartment for 30 min at room temperature prior to perfusing with DPBS and imaging.

### 3.5 Cell viability analysis of μHuB

LIVE/DEAD™ Cell Vitality Assay Kit, C_12_ Resazurin/SYTOX™ Green was used to assess cell viability under static culture, after conditioning to flow, and after dextran transport. Briefly, 10 nM of Sytox green and 500 nM of C_12_‐resazurin was injected in the device. The device was allowed to incubate at 37 °C, 5% CO_2_ for 15 min prior to imaging directly. To determine brain endothelial cell monolayer viability of μHub at the desired probe concentrations, cell vitality assays were performed on post‐ramped cells after 6 hr of constant flow with cell culture media and after an additional 3 hr of flow with 70 kDa nonfluorescent dextran solution (312.5 nM).

### 3.6 Tight junction protein characterization in μHuB (ZO‐1, Claudin‐5)

After flow‐conditioning, μHuB was perfused with DPBS to replace the cell culture media. 4% PFA was injected into all device compartments and allowed to remain at room temperature for 15 min. The device was again perfused with DPBS to remove any residual PFA. Fixed cells were then permeabilized using 0.2% Triton X‐100 in DPBS for 10 min. The device was again perfused with DPBS to move any residual Triton X‐100. The device was blocked with 5% donkey serum and 5% goat serum for 30 min at room temperature. ZO‐1 (1:100) and Claudin‐5 (1:200) primary antibodies were diluted in antibody diluting buffer (0.1% Tween‐20 and 0.1% BSA) at 4 °C overnight. Corresponding fluorescently labeled secondary antibodies Anti‐Goat and Anti‐Donkey (1:1000) was allowed to incubate for 1 hr at room temperature prior to perfusing with DPBS and was immediately imaged.

### 3.7 Acquisition of transport information in μHuB

Following flow‐conditioning, 312.5 nM of FITC‐Dextran (10 and 70 kDa) was injected into the apical channel at 5 μL/min over 2 hr. Device was maintained humidified and at 37 °C and 5% CO_2_ using a Zeiss environmental enclosure. Images were acquired using a 5X objective in 1 min intervals for the duration of the experiment.

### 3.8 Quantification of FITC‐dextran permeation using fluorescent microscopy

Acquired fluorescent image stacks from transport experiments were imported into MATLAB and analyzed using a custom code. Briefly, the average pixel intensity and standard deviation within the apical channel and the basolateral chambers were calculated for each frame. Intensity in the basolateral chamber was normalized to the equilibrium intensity of the apical channel, resulting in a normalized intensity profile (Figure 6c). Frames collected prior to the apical chamber reaching an equilibrium intensity were excluded from the analysis. Permeability was calculated from the normalized intensity profiles using:

(1) $$P=\left(\frac{V}{S}\right)\frac{\mathrm{dI}}{\mathrm{dt}}$$

where *V*/S is the ratio of apical volume to surface area. The linear portion of the resulting intensity over time curve was fit to a line using the MATLAB fit function and weighting with the standard deviations of the intensity. The slope of this line was then used to calculate the permeability as shown in Equation 1 and as described in previous work.^40^, ^81^ Stationary and inflection points were identified using quadratic and cubic fits, respectively, with identical weighting. The permeability of the analyte was assessed by using frames acquired before the intensity profile plateaued. For example, Figure 6d shows a normalized intensity profile for 70 kDa dextran. As before, frames collected prior to the apical chamber reaching its equilibrium value are not included. The profile plateaus between *t* = 50 min and *t* = 100 min. Based on the fitting inflection points, this curve changes slopes at *t* = 60 min. Only frames before *t* = 60 min were used for the permeability calculations. Permeability of the acellular scaffold (*P*_scaffold_) was subtracted from the overall permeability observed (*P*_total_) to calculate the true permeability of the endothelial cell barrier (*P*_e_) for a given tracer (Equation 2).^82^

(2) $$\frac{1}{P_{e}}=\frac{1}{P_{\text{total}}}-\frac{1}{P_{\text{scaffold}}}\;$$

### 3.9 Statistical analysis

Experiments were run in triplicate, and permeability error bars represent a 95% confidence interval based on the linear fitting.

## References

[1] Abbott NJ . Blood–brain barrier structure and function and the challenges for CNS drug delivery. J Inherit Metab Dis. 2013;36(3):437‐449. 10.1007/s10545-013-9608-0. 23609350

[2] Reese TS , Karnovsky MJ . Fine structural localizatin of blood–brain barrier to exogenous peroxidase. J Cell Biol. 1967;34(1):207‐217. 10.1083/jcb.34.1.207. 6033532 PMC2107213

[3] Brightman MW , Reese TS . Junctions between intimately opposed cell membranes in the vertebrate brain. J Cell Biol. 1969;40:648‐677. 10.1083/JCB.40.3.648. 5765759 PMC2107650

[4] Pardridge WM . Drug transport across the blood–brain barrier. J Cereb Blood Flow Metab. 2012;32(11):1959‐1972. 10.1038/jcbfm.2012.126. 22929442 PMC3494002

[5] Cecchelli R , Berezowski V , Lundquist S , et al. Modelling of the blood–brain barrier in drug discovery and development. Nat Rev Drug Discov. 2007;6(8):650‐661. 10.1038/nrd2368. 17667956

[6] Ulapane KR , On N , Kiptoo P , Williams TD , Miller DW , Siahaan TJ . Improving brain delivery of biomolecules via BBB modulation in mouse and rat: detection using MRI, NIRF, and mass spectrometry. Nanotheranostics. 2017;1(2):217‐231. 10.7150/ntno.19158. 28890866 PMC5588751

[7] Banks WA . Mouse models of neurological disorders: a view from the blood–brain barrier. Biochim Biophys Acta Mol Basis Dis. 2010;1802(10):881‐888. 10.1016/j.bbadis.2009.10.011. PMC2891624 19879356

[8] Wohlfart S , Gelperina S , Kreuter J . Transport of drugs across the blood–brain barrier by nanoparticles. J Control Release. 2012;161(2):264‐273. 10.1016/j.jconrel.2011.08.017. 21872624

[9] Mcgonigle P , Ruggeri B . Animal models of human disease: challenges in enabling translation (McGonigle and Ruggeri). Biochem Pharmacol. 2014;87(1):162‐171. 10.1016/j.bcp.2013.08.006. 23954708

[10] Kafkafi N , Agassi J , Chesler EJ , et al. Reproducibility and replicability of rodent phenotyping in preclinical studies. Neurosci Biobehav Rev. 2018;87(October 2016):218‐232. 10.1016/j.neubiorev.2018.01.003. 29357292 PMC6071910

[11] Dash AK , Elmquist WF . Separation methods that are capable of revealing blood–brain barrier permeability. J Chromatogr B Anal Technol Biomed Life Sci. 2003;797(1–2):241‐254. 10.1016/S1570-0232(03)00605-6. 14630153

[12] Passeleu‐Le Bourdonnec C , Carrupt PA , Scherrmann JM , Martel S . Methodologies to assess drug permeation through the blood–brain barrier for pharmaceutical research. Pharm Res. 2013;30(11):2729‐2756. 10.1007/s11095-013-1119-z. 23801086

[13] Pardridge WM . Transport of small molecules through the blood–brain barrier: biology and methodology. Adv Drug Deliv Rev. 1995;15(1–3):5‐36. 10.1016/0169-409X(95)00003-P. 35524389

[14] Patabendige A , Skinner RA , Abbott NJ . Establishment of a simplified in vitro porcine blood–brain barrier model with high transendothelial electrical resistance. Brain Res. 2013;1521:1‐15. 10.1016/j.brainres.2012.06.057. 22789905 PMC3694297

[15] Nakagawa S , Deli MA , Nakao S , et al. Pericytes from brain microvessels strengthen the barrier integrity in primary cultures of rat brain endothelial cells. Cell Mol Neurobiol. 2007;27(6):687‐694. 10.1007/s10571-007-9195-4. 17823866 PMC11517186

[16] Nakagawa S , Deli M a , Kawaguchi H , et al. A new blood–brain barrier model using primary rat brain endothelial cells, pericytes and astrocytes. Neurochem Int. 2009;54:253‐263. 10.1016/j.neuint.2008.12.002. 19111869

[17] Hatherell K , Couraud PO , Romero IA , Weksler B , Pilkington GJ . Development of a three‐dimensional, all‐human in vitro model of the blood–brain barrier using mono‐, co‐, and tri‐cultivation Transwell models. J Neurosci Methods. 2011;199(2):223‐229. 10.1016/j.jneumeth.2011.05.012. 21609734

[18] Freese C , Reinhardt S , Hefner G , Unger RE , Kirkpatrick CJ , Endres K . A novel blood–brain barrier co‐culture system for drug targeting of Alzheimer's disease: establishment by using acitretin as a model drug. PLoS One. 2014;9(3):1‐11. 10.1371/journal.pone.0091003. PMC3946622 24608847

[19] Banks WA , Gray AM , Erickson MA , et al. Lipopolysaccharide‐induced blood–brain barrier disruption: roles of cyclooxygenase, oxidative stress, neuroinflammation, and elements of the neurovascular unit. J Neuroinflammation. 2015;12(1):1‐15. 10.1186/s12974-015-0434-1. PMC4660627 26608623

[20] Helms HC , Abbott NJ , Burek M , et al. In vitro models of the blood–brain barrier: an overview of commonly used brain endothelial cell culture models and guidelines for their use. J Cereb Blood Flow Metab. 2016;36(5):862‐890. 10.1177/0271678X16630991. 26868179 PMC4853841

[21] Madara JL . Regulation of the movement of solutes across tight junctions. Annu Rev Physiol. 1998;60:143‐159. 10.1146/annurev.physiol.60.1.143. 9558458

[22] Gomes MJ , Mendes B , Martins S , Sarmento B . Cell‐based in vitro models for studying blood–brain barrier (BBB) permeability In: Sarmento B, ed, Concepts and Models for Drug Permeability Studies: Cell and Tissue Based In Vitro Culture Models. Cambridge, UK: Woodhead Publishing; 2016;169‐188. 10.1016/B978-0-08-100094-6.00011-0.

[23] Srinivasan B , Kolli AR , Esch MB , Abaci HE , Shuler ML , Hickman JJ . TEER measurement techniques for in vitro barrier model systems. J Lab Autom. 2015;20(2):107‐126. 10.1177/2211068214561025. 25586998 PMC4652793

[24] Davies PF . How do vascular endothelial cells respond to flow? Phys Ther. 1989;4(1):22‐25. http://physiologyonline.physiology.org/content/4/1/22.short.

[25] Shemesh J , Jalilian I , Shi A , Heng Yeoh G , Knothe Tate ML , Ebrahimi Warkiani M . Flow‐induced stress on adherent cells in microfluidic devices. Lab Chip. 2015;15(21):4114‐4127. 10.1039/c5lc00633c. 26334370

[26] Davies PF. Flow‐mediated Mechanotransduction. Physiol Rev 1995;75(3):519–560. doi:10.1152/physrev.1995.75.3.519, Flow‐mediated endothelial mechanotransduction 7624393 PMC3053532

[27] Naik P , Cucullo L . In vitro blood–brain barrier models: current and perspective technologies. J Pharm Sci. 2012;101(4):1337‐1354. 10.1002/jps.23022. 22213383 PMC3288147

[28] Bogorad MI , Destefano J , Karlsson J , Wong AD , Gerecht S , Searson PC . Review: in vitro microvessel models. Lab Chip. 2015;15(22):4242‐4255. 10.1039/C5LC00832H. 26364747 PMC9397147

[29] Cucullo L , Hossain M , Puvenna V , Marchi N , Janigro D . The role of shear stress in blood–brain barrier endothelial physiology. BMC Neurosci. 2011;12:40 10.1186/1471-2202-12-40. 21569296 PMC3103473

[30] Chien S . Mechanotransduction and endothelial cell homeostasis: the wisdom of the cell. AJP Hear Circ Physiol. 2006;292(3):H1209‐H1224. 10.1152/ajpheart.01047.2006. 17098825

[31] Reinitz A , Destefano J , Ye M , Wong AD , Searson PC . Human brain microvascular endothelial cells resist elongation due to shear stress. Microvasc Res. 2015;99:8‐18. 10.1016/j.mvr.2015.02.008. 25725258 PMC4426013

[32] Ye M , Sanchez HM , Hultz M , et al. Brain microvascular endothelial cells resist elongation due to curvature and shear stress. Sci Rep. 2015;4(1):4681 10.1038/srep04681. PMC3986701 24732421

[33] Garcia‐Polite F , Martorell J , Del Rey‐Puech P , et al. Pulsatility and high shear stress deteriorate barrier phenotype in brain microvascular endothelium. J Cereb Blood Flow Metab. 2017;37(7):2614‐2625. 10.1177/0271678X16672482. 27702879 PMC5531355

[34] Gupta N , Liu JR , Patel B , Solomon DE , Vaidya B , Gupta V . Microfluidics‐based 3D cell culture models: utility in novel drug discovery and delivery research. Bioeng Transl Med. 2016;1(1):63‐81. 10.1002/btm2.10013. 29313007 PMC5689508

[35] Jarvis M , Arnold M , Ott J , Pant K , Prabhakarpandian B , Mitragotri S . Microfluidic co‐culture devices to assess penetration of nanoparticles into cancer cell mass. Bioeng Transl Med. 2017;2(3):268‐277. 10.1002/btm2.10079. 29313036 PMC5689499

[36] Booth R , Kim H . Characterization of a microfluidic in vitro model of the blood–brain barrier (μBBB). Lab Chip. 2012;12(10):1784‐1792. 10.1039/c2lc40094d. 22422217

[37] Cucullo L , Couraud P‐O , Weksler B , et al. Immortalized human brain endothelial cells and flow‐based vascular modeling: a marriage of convenience for rational neurovascular studies. J Cereb Blood Flow Metab. 2008;28(2):312‐328. 10.1038/sj.jcbfm.9600525. 17609686

[38] Brown JA , Pensabene V , Markov DA , et al. Recreating blood–brain barrier physiology and structure on chip: a novel neurovascular microfluidic bioreactor. Biomicrofluidics. 2015;9(5):054124 10.1063/1.4934713. 26576206 PMC4627929

[39] Griep LM , Wolbers F , De Wagenaar B , et al. BBB ON CHIP: microfluidic platform to mechanically and biochemically modulate blood–brain barrier function. Biomed Microdevices. 2013;15:145‐150. 10.1007/s10544-012-9699-7. 22955726

[40] Deosarkar SP , Prabhakarpandian B , Wang B , Sheffield JB , Krynska B , Kiani MF . A novel dynamic neonatal blood–brain barrier on a chip. PLoS One. 2015;10(11):1‐21. 10.1371/journal.pone.0142725. PMC4640840 26555149

[41] Walter FR , Valkai S , Kincses A , et al. A versatile lab‐on‐a‐chip tool for modeling biological barriers. Sens Actuators B. 2016;222:1209‐1219. 10.1016/j.snb.2015.07.110.

[42] Dewey CFJ , Bussolari SR , Gimbrone MAJ , Davies PF . The dynamic response of vascular endothelial cells to fluid shear stress. J Biomech Eng. 1981;103(3):177‐185. 10.1115/1.3138276. 7278196

[43] Koutsiaris AG , Tachmitzi S V , Batis N , Kotoula MG , Karabatsas CH , Tsironi E , Chatzoulis DZ Volume flow and wall shear stress quantification in the human conjunctival capillaries and post‐capillary venules in vivo. Biorheology 2007;44(5–6):375–386. doi:N/A 18401076

[44] Modarres HP , Janmaleki M , Novin M , et al. In vitro models and systems for evaluating the dynamics of drug delivery to the healthy and diseased brain. J Control Release. 2018;273(February):108‐130. 10.1016/j.jconrel.2018.01.024. 29378233

[45] Halldorsson S , Lucumi E , Gómez‐Sjöberg R , Fleming RMT . Advantages and challenges of microfluidic cell culture in polydimethylsiloxane devices. Biosens Bioelectron. 2015;63:218‐231. 10.1016/j.bios.2014.07.029. 25105943

[46] Lin K , Hsu PP , Chen BP , et al. Molecular mechanism of endothelial growth arrest by laminar shear stress. Proc Natl Acad Sci USA. 2000;97(17):9385‐9389. 10.1073/pnas.170282597. 10920209 PMC16873

[47] Ziegler T , Nerem RM . Effect of flow on the process of endothelial cell division. Arterioscler Thromb. 1994;14(4):636‐643. http://www.ncbi.nlm.nih.gov/pubmed/8148361. 8148361 10.1161/01.atv.14.4.636

[48] Herculano‐Houzel S . The human brain in numbers: a linearly scaled‐up primate brain. Front Hum Neurosci. 2009;3(November):1‐11. 10.3389/neuro.09.031.2009. 19915731 PMC2776484

[49] Brown NMO , Pfau SJ , Gu C . Bridging barriers: a comparative look at the blood – brain barrier across organisms. 2018;32:466‐478. 10.1101/gad.309823.117.ripherally. PMC5959231 29692355

[50] Herland A , van der Meer AD , Fitzgerald EA , Park T‐E , Sleeboom JJF , Ingber DE . Distinct contributions of astrocytes and Pericytes to Neuroinflammation identified in a 3D human blood–brain barrier on a Chip. PLoS One. 2016;11(3):e0150360 10.1371/journal.pone.0150360. 26930059 PMC4773137

[51] Young EWK , Beebe DJ . Fundamentals of microfluidic cell culture in controlled microenvironments. Chem Soc Rev. 2010;39(3):1036‐1048. 10.1039/b909900j. 20179823 PMC2967183

[52] Helms HC . In vitro models of the blood–brain barrier; an overview of commonly used brain endothelial cell culture models and guidelines for their use. J Cereb Blood Flow Metab. 2016;36:862‐890. 10.1177/0271678X16630991. 26868179 PMC4853841

[53] Young EWK , Wheeler AR , Simmons CA . Matrix‐dependent adhesion of vascular and valvular endothelial cells in microfluidic channels. Lab Chip. 2007;7(12):1759‐1766. 10.1039/b712486d. 18030398

[54] Wang X , Phan DTT , Sobrino A , George SC , Hughes CCW , Lee AP . Engineering anastomosis between living capillary networks and endothelial cell‐lined microfluidic channels. Lab Chip. 2016;16(2):282‐290. 10.1039/C5LC01050K. 26616908 PMC4869859

[55] Weksler BB , Subileau EA , Perrie N , et al. Blood–brain barrier‐specific properties of a human adult brain endothelial cell line. FASEB J. 2005;19(13):1872‐1874. 10.1096/fj.04-3458fje. 16141364

[56] Dauchy S , Miller F , Couraud PO , et al. Expression and transcriptional regulation of ABC transporters and cytochromes P450 in hCMEC/D3 human cerebral microvascular endothelial cells. Biochem Pharmacol. 2009;77(5):897‐909. 10.1016/j.bcp.2008.11.001. 19041851

[57] Weksler B , Romero IA , Couraud P‐O . The hCMEC/D3 cell line as a model of the human blood brain barrier. Fluids Barriers CNS. 2013;10(1):16 10.1186/2045-8118-10-16. 23531482 PMC3623852

[58] Poller B , Gutmann H , Krähenbühl S , et al. The human brain endothelial cell line hCMEC/D3 as a human blood–brain barrier model for drug transport studies. J Neurochem. 2008;107(5):1358‐1368. 10.1111/j.1471-4159.2008.05730.x. 19013850

[59] Rahman NA , Rasil ANHM , Meyding‐Lamade U , et al. Immortalized endothelial cell lines for in vitro blood–brain barrier models: a systematic review. Brain Res. 2016;1642:532‐545. 10.1016/j.brainres.2016.04.024. 27086967

[60] Tanzeglock T , Soos M , Stephanopoulos G , Morbidelli M . Induction of mammalian cell death by simple shear and extensional flows. Biotechnol Bioeng. 2009;104(2):360‐370. 10.1002/bit.22405. 19575444

[61] Fulda S , Gorman AM , Hori O , Samali A . Cellular stress responses: cell survival and cell death. Int J Cell Biol. 2010;2010:1‐23. 10.1155/2010/214074. PMC2825543 20182529

[62] Kadohama T , Nishimura K , Hoshino Y , Sasajima T , Sumpio BE . Effects of different types of fluid shear stress on endothelial cell proliferation and survival. J Cell Physiol. 2007;212(1):244‐251. 10.1002/jcp.21024. 17323381

[63] Eigenmann DE , Xue G , Kim KS , Moses AV , Hamburger M , Oufir M . Comparative study of four immortalized human brain capillary endothelial cell lines, hCMEC/D3, hBMEC, TY10, and BB19, and optimization of culture conditions, for an in vitro blood–brain barrier model for drug permeability studies. Fluids Barriers CNS. 2013;10(1):33 10.1186/2045-8118-10-33. 24262108 PMC4176484

[64] Weksler B , Romero IA , Couraud P‐O . The hCMEC/D3 cell line as a model of the human blood brain barrier. Fluids Barriers CNS. 2013;10(1):16 10.1186/2045-8118-10-16. 23531482 PMC3623852

[65] Li G , Yuan W , Fu BM . A model for the blood–brain barrier permeability to water and small solutes. J Biomech. 2010;43(11):2133‐2140. 10.1016/j.jbiomech.2010.03.047. 20434157

[66] Cucullo L , Hossain M , Puvenna V , Marchi N , Janigro D . The role of shear stress in blood–brain barrier endothelial physiology. BMC Neurosci. 2011;12:40 10.1186/1471-2202-12-40. 21569296 PMC3103473

[67] Thomsen MS , Routhe LJ , Moos T . The vascular basement membrane in the healthy and pathological brain. J Cereb Blood Flow Metab. 2017;37(10):3300‐3317. 10.1177/0271678X17722436. 28753105 PMC5624399

[68] Hallmann R , Horn N , Selg M , Wendler O , Pausch F , Sorokin LM . Expression and function of Laminins in the embryonic and mature vasculature. Physiol Rev. 2005;85(3):979‐1000. 10.1152/physrev.00014.2004. 15987800

[69] Prabhakarpandian B , Shen M‐C , Nichols JB , et al. SyM‐BBB: a microfluidic blood brain barrier model. Lab Chip. 2013;13(6):1093‐1101. 10.1039/c2lc41208j. 23344641 PMC3613157

[70] Madara JL . Regulation of the movement of solutes across tight junctions. Annu Rev Physiol. 1998;60(1):143‐159. 10.1146/annurev.physiol.60.1.143. 9558458

[71] Abbott NJ , Patabendige AAK , Dolman DEM , Yusof SR , Begley DJ . Structure and function of the blood–brain barrier. Neurobiol Dis. 2010;37(1):13‐25. 10.1016/j.nbd.2009.07.030. 19664713

[72] Kawedia JD , Nieman ML , Boivin GP , et al. Interaction between transcellular and paracellular water transport pathways through aquaporin 5 and the tight junction complex. Proc Natl Acad Sci USA. 2007;104(9):3621‐3626. 10.1073/pnas.0608384104. 17360692 PMC1802728

[73] Kaya M , Ahishali B . Assessment of permeability in barrier type of endothelium in brain using tracers: Evans blue, sodium fluorescein, and horseradish peroxidase In: Turksen K, ed. Permeability Barrier: Methods and Protocols. Totowa, NJ: Humana Press; 2011:369‐382. 10.1007/978-1-61779-191-8_25. 21874465

[74] Juliano RL . In: Juliano RL, ed. Targeted Drug Delivery. Vol 100 Berlin, Heidelberg: Springer Berlin Heidelberg; 1991 10.1007/978-3-642-75862-1.

[75] Bouldin TW , Krigman MR . Differential permeability of cerebral capillary and choroid plexus to lanthanum ion. Brain Res. 1975;99(2):444‐448. 10.1016/0006-8993(75)90053-0. 1182566

[76] Cserr HF , Bundgaard M . Blood–brain interfaces in vertebrates: a comparative approach. Am J Physiol. 1984;246(3 Pt 2):R277‐R288. 10.1152/ajpregu.1984.246.3.R277. 6367490

[77] Yuan W , Lv Y , Zeng M , Fu BM . Non‐invasive measurement of solute permeability in cerebral microvessels of the rat. Microvasc Res. 2009;77(2):166‐173. 10.1016/j.mvr.2008.08.004. 18838082

[78] Sajja RK , Prasad S , Cucullo L . Impact of altered glycaemia on blood–brain barrier endothelium: an in vitro study using the hCMEC/D3 cell line. Fluids Barriers CNS. 2014;11(1):8‐237. 10.1007/s10836-006-7823-4. 24708805 PMC3985548

[79] Rubin LL . A cell culture model of the blood–brain barrier. J Cell Biol. 1991;115(6):1725‐1735. 10.1083/jcb.115.6.1725. 1661734 PMC2289219

[80] von Bartheld CS , Bahney J , Herculano‐Houzel S . The search for true numbers of neurons and glial cells in the human brain: a review of 150 years of cell counting. J Comp Neurol. 2016;524(18):3865‐3895. 10.1002/cne.24040. 27187682 PMC5063692

[81] Yuan H , Gaber MW , McColgan T , Naimark MD , Kiani MF , Merchant TE . Radiation‐induced permeability and leukocyte adhesion in the rat blood–brain barrier: modulation with anti‐ICAM‐1 antibodies. Brain Res. 2003;969:59‐69. 10.1016/S0006-8993(03)02278-9. 12676365

[82] Dehouck M‐P , Jolliet‐Riant P , Brée F , Fruchart J‐C , Cecchelli R , Tillement J‐P . Drug transfer across the blood–brain barrier: correlation between in vitro and in vivo models. J Neurochem. 1992;58(5):1790‐1797. 10.1111/j.1471-4159.1992.tb10055.x. 1560234

